# Spatiotemporal Facility‐Level Patterns of Summer Heat Exposure, Vulnerability, and Risk in United States Prison Landscapes

**DOI:** 10.1029/2024GH001108

**Published:** 2024-09-24

**Authors:** Ufuoma Ovienmhada, Mia Hines, Michael Krisch, Ahmed T. Diongue, Brent Minchew, Danielle R. Wood

**Affiliations:** ^1^ Department of Aeronautics and Astronautics Massachusetts Institute of Technology Cambridge MA USA; ^2^ Department of Computer Science Grinnell College Grinnell IA USA; ^3^ Brown Institute for Media Innovation Columbia University New York NY USA; ^4^ Department of Earth, Atmospheric, and Planetary Sciences Massachusetts Institute of Technology Cambridge MA USA; ^5^ Space Enabled Research Group Massachusetts Institute of Technology Cambridge MA USA

**Keywords:** carceral ecology, geospatial data, toxic prisons, environmental justice, extreme heat, heat risk

## Abstract

Heat is associated with increased risk of morbidity and mortality. People who are incarcerated are especially vulnerable to heat exposure due to demographic characteristics and their conditions of confinement. Evaluating heat exposure in prisons, and the characteristics of exposed populations and prisons, can elucidate prison‐level risk to heat exposure. We leveraged a high‐resolution air temperature data set to evaluate short and long‐term patterns of heat metrics for 1,614 prisons in the United States from 1990 to 2023. We found that the most heat‐exposed facilities and states were mostly in the Southwestern United States, while the prisons with the highest temperature anomalies from the historical record were in the Pacific Northwest, the Northeast, Texas, and parts of the Midwest. Prisons in the Pacific Northwest, the Northeast, and upper Midwest had the highest occurrences of days associated with an increased risk of heat‐related mortality. We also estimated differences in heat exposure at prisons by facility and individual‐level characteristics. We found higher proportions of non‐white and Hispanic populations in the prisons with higher heat exposure. Lastly, we found that heat exposure was higher in prisons with any of nine facility‐level characteristics that may modify risk to heat. This study brings together distinct measures of exposure, vulnerability, and risk, which would each inform unique strategies for heat‐interventions. Community leaders and policymakers should carefully consider which measures they want to apply, and include the voices of directly impacted people, as the differing metrics and perspectives will have implications for who is included in fights for environmental justice.

## Key Points

- Prisons, incarcerated populations, and staff are exposed to heat and changing climates as measured through a variety of metrics
- Higher temperatures are found in prison landscapes that have characteristics that can modify exposure or vulnerability, increasing overall risk
- Distinct measures of exposure, vulnerability, and risk, can each inform unique strategies for heat‐interventions in US prisons

## 1 Introduction

Heat exposure is associated with an increased risk of morbidity and mortality (Li et al., 2015; Xu et al., 2016). People who are incarcerated in the United States (US) are especially vulnerable to extreme heat as an increasingly older and disabled population (Anno et al., 2004; Kenny et al., 2010; Skarha et al., 2023). Incarcerated populations also have the added precarity of having their housing, health care, and personal security determined by correctional agencies that may not have adequate infrastructure, policies, staffing, or heat‐intervention resources (Colucci et al., 2023; Enggist et al., 2014; Purdum et al., 2022). For example, while the provision of air conditioning (AC) has been shown to effectively eliminate heat‐related mortality in Texas prisons (Skarha et al., 2022), 44 states including Texas do not universally provide access to air conditioning in prisons (Santucci et al., 2022). Common characteristics of prisons such as overcrowding, poor medical facilities, isolation, and inadequate mental health treatment can all create more risk to extreme heat exposure (Cloud et al., 2023; Holt, 2015; Skarha et al., 2023). Despite the susceptibility of the incarcerated population to the negative impacts of heat exposure, minimal work has been done on heat as an environmental risk factor in prisons.

An analysis of environmental risk typically includes three components: *hazard*—the occurrence of natural or human‐induced physical events that can have adverse effects; *exposure*—the intersection of a hazard and human populations or infrastructure; and *vulnerability*—the propensity of exposed humans or infrastructure to suffer adverse effects upon exposure (Cardona et al., 2012). There is a limited amount of prior work that has touched on these three components needed to assess risk to heat in prisons. A Colorado‐based study and a national study found high and hazardous heat exposure in carceral landscapes (Glade et al., 2024; Tuholske et al., 2024). However, only the Colorado‐based study includes assessment of facility and demographic characteristics that may have vulnerability and equity implications for environmental risk. Skarha et al. (2023) found that higher exposure to heat is associated with increases in mortality risk nationally across US state and private prisons, and that several regional and demographic characteristics increase vulnerability and risk. However, this study only assesses two facility‐level characteristics (security level and jurisdiction) that could relate to the vulnerability of individual prisons to heat exposure. Furthermore, all prior studies on spatiotemporal patterns of heat exposure or vulnerability conclude in or prior to 2020, which excludes two of the seven hottest years on record for the United States (National Centers for Environmental Information, 2024). There is a need for more research evaluating the spatiotemporal patterns of historical contemporary heat exposure and the facility or individual‐level characteristics of exposed prisons and incarcerated populations. This type of research can elucidate prison‐level risk to heat exposure, which has implications for informing policy and other interventions.

We build on the aforementioned research by presenting a national‐scale assessment on the relationship between prisons, heat exposure, individual‐level characteristics, and facility‐level characteristics that could modify risk to heat exposure. First, we use a high‐resolution modeled air temperature data set to characterize the short (2020–2023) and long‐term (1990–2023) spatial pattern of air temperature at individual prisons and by state. We also estimate facility‐level risk of heat‐related mortality from 2020–2023 based on previous epidemiological findings. Lastly, we estimate differences in heat exposure at prisons by individual and facility‐level characteristics for the year 2019, which sheds light on population groups that may experience elevated heat risk. By assessing spatiotemporal patterns of heat exposure through short and long‐term patterns, and the related lens of risk, our study shows a variety of ways that addressing hazardous heat in prisons could be prioritized and acted upon. We anticipate that our study can be used to inform mitigation approaches aimed at reducing heat exposure in prisons, with a consideration of facility‐level risk to adverse effects from heat for the incarcerated population.

## 2 Materials and Methods

### 2.1 Prison Facility and Demographic Data

Information on the location and boundaries of carceral facilities in the US were obtained from the Homeland Infrastructure Foundation Level Data (HIFLD) database maintained by the Department of Homeland Security (US Department of Homeland Security, 2022). We used the shapefile version of the data set which includes polygon boundaries of prisons, jails, and detention centers that range in jurisdiction from federal to local governments. The data set was filtered to only include facilities under state, federal, multi, or unknown jurisdictions that were open as of June 2020. We excluded juvenile facilities and other carceral facilities that were deemed to be different from prisons (i.e., either in function, population or infrastructure). The final data set included 1,614 facilities that we describe as prisons here. Additional information about this data set is available at (Ovienmhada et al., 2024).

Demographic and facility‐level characteristics of prisons were obtained from the Bureau of Justice Statistics. Specifically, we obtained the 2019 “Census of State and Federal Adult Correctional Facilities” (referred to hereafter as “BJSCensus2019”) data set (Maruschak & Buehler, 2021). The BJSCensus2019 includes 271 attributes for individual prisons on incarcerated and staff population and demographics, facility functions, prison labor activities, and conditions of confinement. Using HIFLD as the more comprehensive source of facility locations, we merged this data set with the detailed sociodemographic and facility‐level data available in the BJS Census2019. A total of 1,292 (80%) of records in the HIFLD data set matched with the BJS Census2019. Table S1 in Supporting Information S1 provides a summary of all of the variables from BJSCensus2019 that were linked to HIFLD polygons. The approach is described in detail in Ovienmhada et al. (2024). Figure S1 in Supporting Information S1 provides a map of prisons that were not matched to the census data. For facilities with non‐matching BJSCensus2019 data (20%), we used the HIFLD population data when discussing overall prison population totals but omit those prisons from the statistical analysis of facility‐level or individual characteristics.

### 2.2 Temperature Data

We obtained daily outdoor air temperatures on a 1‐km grid for 1990–2023 from the freely available Daymet v4 Surface Weather and Climatological summaries (M. M. Thornton et al., 2022). The start year of 1990 was chosen because the majority (∼59%) of state and federal correctional facilities were built by 1990 and 82% were built by 1999 (Stephan, 2008). We chose ambient air temperature as our heat exposure variable as it has been extensively shown in the literature to be related to adverse heat‐related health outcomes (Hajat & Kosatky, 2010; Medina‐Ramón & Schwartz, 2007). In the Discussion section, we address the potential relevance of other exposure variables. Daymet also has the advantage of being available at higher spatial resolution and for more recent years than other modeled temperature data (such as NLDAS or PRISM, respectively), which is important for characterizing heat exposure more granularly and in light of warming trends. Validation and accuracy assessment of the Daymet air temperature model is available at P. E. Thornton et al. (2021).

The data was filtered to summer months defined here as 1 June through 31 August. We then calculated four heat exposure metrics at individual prisons from the daily summer air temperature measurements averaged for the years 2020–2023: (a) the number of days the daily mean temperature is greater than 85°F, (b) the 90th percentile of daily max air temperature, (c) the anomaly in average maximum summer temperatures, and (d) the number of days a prison experiences a temperature 10° higher than their prison location‐specific mean summer temperature. The first metric was chosen because while policies related to thermoregulation in prisons are far from universal, carceral systems that do have temperature policies commonly regulate that the upper limit of air temperature remain below 85°F (Holt, 2015). This metric thus illustrates where prison daily mean temperatures are in excess of a plausible standard supported by some community‐based advocacy organizations such as the Texas Prison Community Advocates and Lioness. The second metric was used to represent location‐specific extreme temperatures. The third metric, anomaly, is defined here as the difference between a 4‐year summer average 90th percentile temperature (2020–2023) and a 30‐year historical (1990–2019) summer average 90th percentile temperature. Lastly, the fourth metric was associated with a 5.2% increase in total mortality across state and private prisons nationwide from 2001 to 2019 (Skarha et al., 2023) and we used it here as a proxy for estimating where prisons may have experienced increases in risk of heat‐related mortality from 2020 to 2023, years not included in the study.

### 2.3 Heat Exposure by Individual and Facility‐Level Characteristics

To estimate differences in heat exposure at prisons by individual‐level characteristics, we used the 2019 summer 90th percentile of daily max air temperature to match with the year of BJSCensus2019. We excluded prisons in Alaska and Hawaii (*n* = 20) from this portion of the analysis due to relatively homogenous demographics and mild to moderate heat exposures, and we exclude prisons with incomplete race/ethnicity data. There were 1,260 (78.0%) remaining records with complete data on the incarcerated population by race/ethnicity. The BJSCensus2019 categorizes this data under one question with eight categories including “White, not Hispanic,” “Black, not Hispanic,” “Hispanic or Latino,” “American Indian/Alaskan Native, non Hispanic,” “Asian, not Hispanic,” “Native Hawaiian or other Pacific Islander, not Hispanic,” “Two or more races, not Hispanic,” and “Additional Categories” etc. We combined six of the eight categories into a non‐white, non‐Hispanic group. Then, we assessed differences in the 90th percentile heat exposure by race/ethnicity by producing descriptive statistics for (a) prisons with the top and lowest 10 short‐term heat exposures and (b) prisons within the top and lowest decile (*n* = 128) of short‐term heat exposures. Lastly, a mean heat exposure was calculated for each demographic group weighted by the facility‐level populations of a given group. Specifically, population‐weighted heat exposures were calculated as the product of the prison summer 90th percentile temperature (*T*_*j*_) and the demographic group population (*p*_*j*_) in the *i* th prison, summed over all prisons (*n* = 1,614) and divided by the summation of the demographic group population (*p*_*j*_) (Equation 1).

(1) $$\text{population}-\text{weighted}\;T_{j}=\sum_{i=1}^{n}{T_{j}p}_{i,j}/\sum_{i=1}^{n}p_{i,j}$$

Next, we evaluated how heat exposure varies by facility‐level characteristics. We first examined the peer‐reviewed literature to identify characteristics (measured by the BJSCensus2019) that we hypothesized could modify risk to heat exposure either by directly increasing heat exposure or increasing vulnerability. Here, vulnerability could be produced as a result of increased susceptibility to adverse effects due to the characteristics of a group or decreased capacity to cope with adverse effects. A summary of the 17 incorporated facility‐level characteristics and supporting literature are shown in Table 1. The variables related to “Poor Conditions” indicate that a prison was under court order to improve those conditions as of June 2019. For the facility‐level characteristics (e.g., presence or absence of a geriatric unit, poor conditions: crowding) with binary values, we conducted unpaired *t*‐tests to determine if the mean 90th percentile temperature of two groups were different and statistically significant. The Wilcoxon rank‐sum test (i.e. Mann‐Whitney U) was applied instead of a *t*‐test in cases where data were nonparametric and the number of facilities was less than 30 for at least one group. For the facility‐level characteristics with more than two values (e.g., Security Level), we conducted a one‐way analysis of variance or Welch test depending on the variable's distribution. For all statistical analyses, an alpha level of 0.05 was used.

**Table 1:** Facility‐Level Heat Risk Variables and Literature That Support a Relationship Between the Given Variables and Adverse Heat‐Related Health Outcomes

Variable	Values	Hypothesized effect on heat risk	Supporting literature or rationale
Geriatric Unit Present in facility	No, Yes	Increased susceptibility	Benmarhnia et al. (2015), Jones et al. (1982), Kenny et al. (2010), Li et al. (2015), and Whitman et al. (1997)
Poor conditions: crowding	No, Yes	Increased heat exposure; decreased capacity to cope	American Society of Heating and Engineers (1997), Holt (2015), and Purdum et al. (2022)
Poor conditions: mental health treatment	No, Yes	Increased susceptibility	Meadows et al. (2024) and Stivanello et al. (2020)
Poor conditions: medical facilities	No, Yes	Decreased capacity to cope	Li et al. (2015)
Poor conditions: accommodations of the disabled	No, Yes	Decreased capacity to cope; increased susceptibility	Gaskin et al. (2017) and Park et al. (2024)
Poor conditions: disciplinary procedures	No, Yes	Decreased capacity to cope	The incarcerated population may have reduced ability to access heat‐intervention resources due to fear of discipline or retaliation (Ovienmhada et al., 2023; Purdum et al., 2022)
Poor conditions: grievance procedures	No, Yes	Decreased capacity to cope	The incarcerated population may have reduced ability to access heat‐intervention resources due to inadequacies in grievance procedures (Friedman, 2021)
Poor conditions: staffing	No, Yes	Decreased capacity to cope	Facilities with inadequate staff may not be able to regularly administer heat‐intervention procedures (Purdum et al., 2022)
Poor conditions: totality of conditions	No, Yes	Decreased capacity to cope	Supported by aforementioned literature
Inmate Work Assignments in Farming/Agriculture	No, Yes	Increased exposure; increased susceptibility	Uejio et al. (2018) and Venugopal et al. (2021)
Primary facility function: Medical treatment	No, Yes	Increased susceptibility	Facilities performing medical treatments can be at risk to equipment failure from heat (Carmichael et al., 2012)
Primary facility function: Mental Health	No, Yes	Increased susceptibility	Meadows et al. (2024) and Stivanello et al. (2020)
Primary facility function: Geriatric Care	No, Yes	Increased susceptibility	Benmarhnia et al. (2015), Jones et al. (1982), Kenny et al. (2010), Li et al. (2015), and Whitman et al. (1997)
Primary facility function: Boot Camp	No, Yes	Increased exposure; increased susceptibility	Boot camp style prisons often require high physical activity and work requirements which can increase heat stress (Nichols, 2014)
Security Level of Facility	Minimum/low	Decreased capacity to cope	Facilities with higher security levels may reduce the ability of individuals to perform heat‐adaptive behaviors
	Medium		
	Maximum/high		
Percentage of inmates permitted to leave facility	50% or more	Decreased capacity to cope	Facilities with more restriction on movement may reduce the ability of individuals to perform heat‐adaptive behaviors
	Less than 50%		
	None		
Operator of the Facility	Public	Decreased capacity to cope	Private prisons are not subject to the same regulations as government prisons (Vilher, 2017) and thus may have less policies related to heat risk
	Private		

## 3 Results

A total of 1,614 facilities approximated to be prisons in the US were assigned heat exposure metrics (Table 2). Of those, 977 (60.5%) facilities have a minimum or medium security level, while 151 (9.4%) have “close” security which refers to high‐security facilities where movement may be restricted 24 hr a day. The 1,614 facilities incarcerate an estimated 1,392,659 people and have an estimated 337,096 staff. The states with the largest number of facilities were Florida, Texas, and California which had about 29% of the total incarcerated population, slightly higher than their proportion (27.4%) of the total US population in 2019 (Bureau, 2024).

**Table 2:** Overview of Prisons and Their Incarcerated and Staff Populations

	Count or population (mean)
Facilities, *N*	1,614
Facility Operator, *N*	
State	1,370
Federal	220
Multi/Other	24
Security Level of Facility, *N*	
Minimum	558
Medium	419
Maximum	335
Close	151
Incarcerated Population	1,392,659 (1,015)
Staff Population	337,096 (330)

*Note.* These population estimates are a combination of records from BJSCensus2019 (*n* = 1,292) and records from the HIFLD (*n* = 259). Eighty facilities did not have population information available in either data set. Staff population records are only available for BJSCensus2019 records (*n* = 1,292).

**Figure 1:**
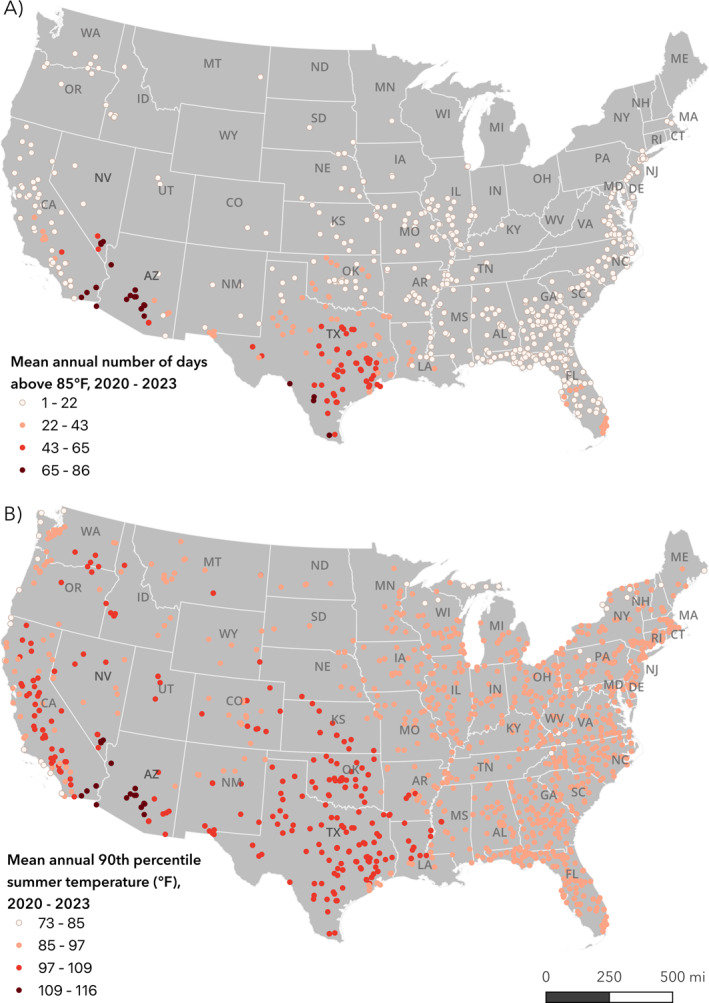
Prisons by (a) annual number of days above 85°F and (b) 90th percentile summer temperature, both averaged 2020–2023.

The majority (67.3%) of facilities (at least 986,145 incarcerated people and 222,154 staff) in 44 states and the District of Columbia had at least one day that the daily mean temperature was over 85°F from 2020 to 2023 (Figure 1a). Prisons in Florida, Texas, California, Georgia, and Illinois accounted for 46.0% of all prisons with this exposure. There were prisons in California, Nevada, Arizona, and Texas that had at least 65 days that the daily mean temperature was over 85°F from 2020 to 2023. Figure 1b shows variation in the location‐specific 90th percentile temperature at prisons averaged from 2020 to 2023. The mean (SD) value for all prisons was 93.6°F (5.8°F). The top 10 most heat‐exposed facilities were in California, Arizona and Nevada (Figure 1b). The facility with the maximum value (116.1°F) was Calipatria State Prison in California which is a facility that has air conditioning in at least some parts of the unit, but it has failed during extremely hot conditions (Maresh, 2021). Until 2021, the prison with the second highest value (114.7°F), Ironwood state prison, only had an “ineffective” and “unreliable” evaporative cooling system for temperature regulation (State of California, 2021). From 2020 to 2023, the mean (SD) summer 90th percentile temperature at carceral facilities averaged state‐wide was 93.1°F (4.5°F) and the max, Arizona, was 109.1°F (Figure S2 in Supporting Information S1). The next highest prison heat exposures averaged state‐wide were in Nevada, Texas, Oklahoma, Utah, Kansas, New Mexico and Louisiana (Figure S2 in Supporting Information S1).

**Figure 2:**
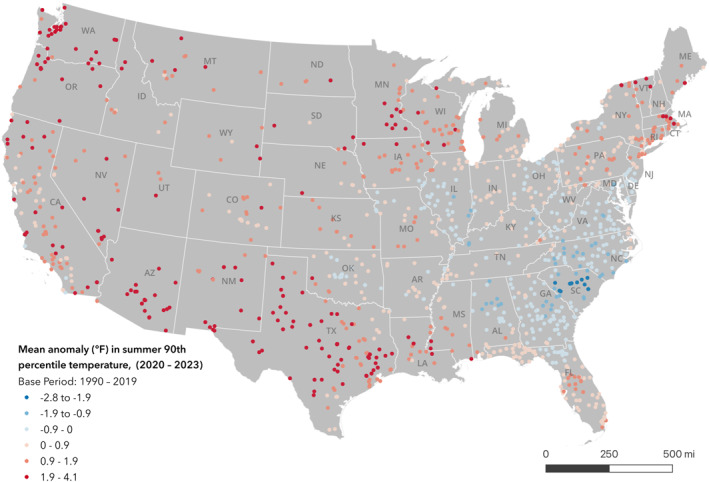
Prisons by mean anomaly (°F) in summer 90th percentile temperature (2020–2023). Anomaly shows the difference from a 1990–2019 average. Equal interval classification scheme used.

Figure 2 shows the spatial pattern of anomalies in the average 90th percentile summer temperature for prisons from 2020 to 2023 when compared to a 1990–2019 averaged summer 90th percentile. From 2020 to 2023, there were positive changes in the upper decile of heat exposure for 1,135 (70.3%) prisons meaning these prisons experienced approximately 9 out of 92 total days of summer that were hotter than all previous summers, on average, for a given location. The mean (SD) anomaly for all prisons was 0.7°F (1.1°F). The highest facility‐level temperature anomalies occurred at prisons in Washington, California, Maine, Idaho, and Texas. The maximum anomaly was 4.1°F which occurred at Washington Corrections Center, a maximum security prison without universal AC that currently incarcerates around 1,700 people, which is above the design capacity of 1,268.

**Figure 3:**
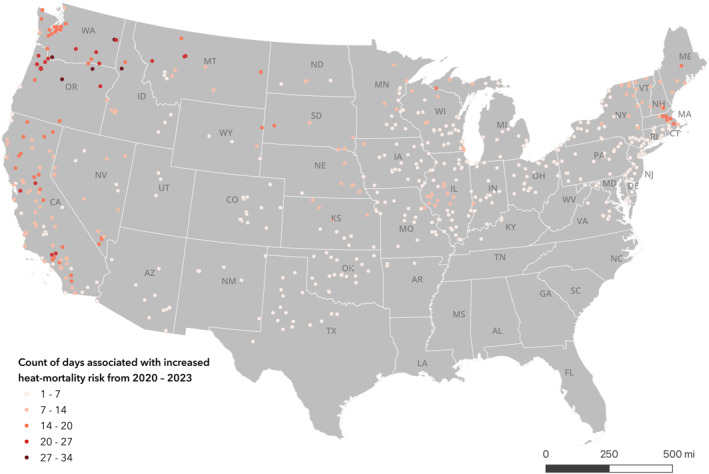
Prisons by count of days associated with increased heat‐mortality risk from 2020 to 2023. A zero count **does not imply** that heat‐related deaths did not occur. Rather, those prisons may be experiencing regularly high counts of deaths in regularly high temperatures that do not deviate from the average in those climates.

From 2020 to 2023, 828 (51.3%) facilities had at least one day that the prison experienced an outdoor temperature 10°F higher than their location‐specific mean summer temperature (Figure 3). Each of these days may be associated with a 5.2% increase in total mortality for the prison population, on average (Skarha et al., 2023). A count of zero does not imply that heat‐related deaths did not occur. Rather, those places may be experiencing regularly high counts of deaths in regularly high temperatures that do not deviate from the average in those climates. Higher counts were apparent in the Northeast and Pacific Northwest. The mean (SD) number of days 10°F above prison mean was 3.5 days (5.4 days) and the max was 34 days. Facilities with the highest number Days 10°F above prison mean include Deer Ridge Correctional Institution in Oregon, Larch Corrections Center in Washington, and North Idaho Correctional Institution. Figure 3 shows that both the spatial pattern of the highest heat exposures (Figure 1b) and exposures above a plausibly “safe” temperature of 85°F (Figure 1a) differ from the spatial pattern of areas that may have elevated heat‐related mortality risk based on this climate‐specific metric.

**Figure 4:**
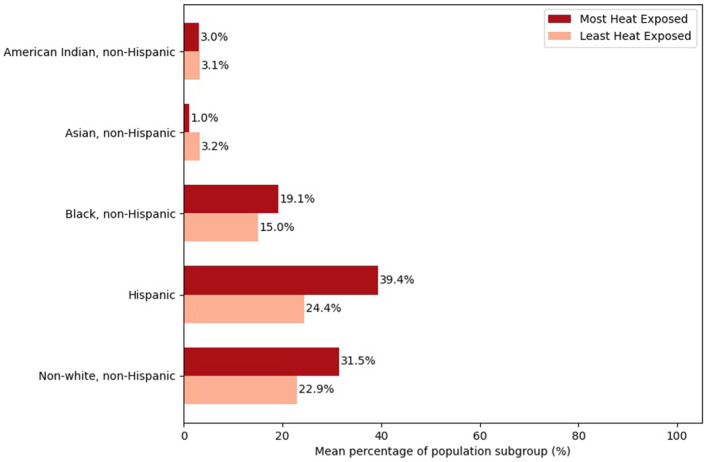
Proportion of select demographic subgroups in the top and bottom 10 most heat exposed prisons.

In 2019, the incarcerated populations of different race/ethnicity groups were unequally exposed to higher heat (as measured by the 90th percentile summer temperature) in prison landscapes (Tables S2 and S3 in Supporting Information S1). Across the US, the prisons with the top 10 heat exposures had 8.6% higher proportions of nonwhite, non‐Hispanic incarcerated populations than the lowest 10 prisons (Figure 4). This difference was driven largely by the Black, non‐Hispanic subgroup (+4.1%) representation in the most heat‐exposed prisons. The prisons in the top and lowest 10 are all in states that have disproportionately higher incarceration rates for people of color than for the white, non‐Hispanic group (Prison Policy Initiative, 2024). However, the state for two of the prisons in the lowest 10 (Oregon) have higher proportions of white, non‐Hispanic people in general, which may be contributing to the disparity in exposure (World Population Review, 2024). The prisons with the top 10 heat exposures also had 15.0% higher proportions of Hispanic or Latino incarcerated populations than the lowest 10 prisons (Figure 4). When examining population subgroups for the prisons in the top and lowest decile (*n* = 126) of heat exposure, only the Hispanic subgroup had a higher incarcerated proportion (+26.0%) in the top heat exposure decile (Table S3 in Supporting Information S1). Across all prisons, the population‐weighted summer heat exposure for the white, non‐Hispanic, Black, non‐Hispanic, American Indian non‐Hispanic, Asian, non‐Hispanic, and non‐white, non‐Hispanic incarcerated populations was between 93.2 and 94.7°F, while the Hispanic or Latino subgroup had the highest population‐weighted exposure at 97.1°F (Table S4 in Supporting Information S1).

**Figure 5:**
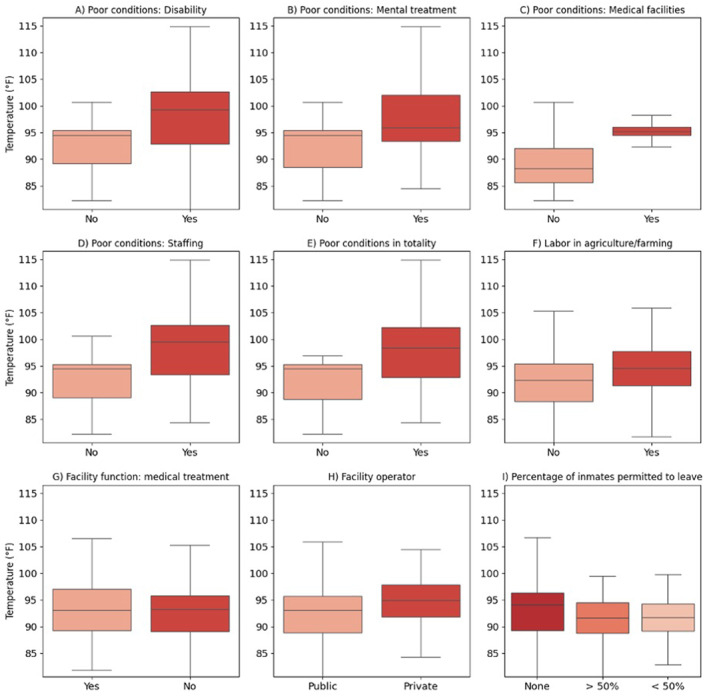
Prison heat exposures by nine facility‐level characteristics that could modify heat risk. Boxes depict temperature interquartile range for each facility‐level characteristic group. The middle line within the box marks the median temperature. Differences are statistically significant.

Next, we assessed how heat exposure (as measured by the 90th percentile summer temperature in 2019) is distributed by facility‐level characteristics related to facility operations, housing, or conditions in US prisons (see Table 1) that we hypothesize could modify risk to heat exposure (Figure 5). Nine of the 17 test variables had statistically significant (*p* < 0.05) higher heat exposures for the groups with hypothesized effects on heat risk suggesting that these groups may face elevated risk not only due to these facility‐level characteristics but also due to elevated heat exposures. This includes prisons that had a primary facility function of providing medical treatment. Prisons that were under court order as of June 2019 for poor conditions of confinement including mental health services, medical facilities, staffing, accommodation of the disabled, and totality of conditions, all had higher (*p* < 0.05) heat exposures than prisons without those conditions. Prisons that have work assignments in agriculture or farming had higher (*p* < 0.05) heat exposures than prisons without that type of labor activity.

We also found that heat exposure differed (*p* < 0.001) based on the facility operator and the percentage of incarcerated people permitted to regularly leave the facility for work or study. Post‐hoc tests found that prisons that were operated privately had higher (+2.7°F) heat exposure than public facilities. Lastly, prisons that do not permit any of the incarcerated population to leave the premises for work or study had higher (+2.0°F) heat exposure than facilities where the majority of the population is permitted to leave the facility. We did not find evidence that heat exposure significantly differed based on the security level of a facility, conditions of confinement related to crowding or grievance procedures, or prisons with primary facility functions in geriatric care or alcohol/drug treatment. Complete information on statistical tests for all variables are included in Tables S5 and S6 in Supporting Information S1.

## 4 Discussion

Our study leveraged a high‐resolution temperature data set to examine spatiotemporal patterns of heat exposure and risk in US prison landscapes in four ways not previously done in the literature and for more recent years than previous work. With regards to the patterns of the 90th percentile summer air temperature, we found that the most heat‐exposed facilities and the states with the highest prison heat exposures on average were mostly in the Southwestern US where there are not universal air conditioning regulations in prisons (Santucci et al., 2022). However, most (98.2%) of all prisons in the US are experiencing an upper decile of summer temperatures that exceed the upper bound of temperatures that several health organizations and governments advise temperatures should stay below for thermal comfort (Kenny et al., 2019). Relatedly, we found that over two‐thirds of all state and federal prisons had at least one day when the external air temperature was above 85°F which is an upper limit commonly used by the subset of jurisdictions that have standards related to thermoregulation in carceral facilities (Holt, 2015). Then, we assessed heat exposure based on temperature anomalies relative to a historical baseline which enabled assessment of warming trends specific to a prison's geographic location. With this approach, we found that the highest facility‐level temperature anomalies from 2020 to 2023 occurred in the Pacific Northwest, the Northeast, Texas, and parts of the Midwest. Some of this pattern aligns with the regional warming trends identified in broader climate studies (Karmalkar & Bradley, 2017; Vose et al., 2017). We also assessed the spatial pattern of a heat exposure metric associated with an increase in mortality risk in US prisons (Skarha et al., 2023). We found that prisons in the Pacific Northwest, the Northeast, upper Midwest, and California had the highest occurrences of this health‐relevant heat exposure metric. This is especially concerning given that Skarha et al. (2023) found that the risk ratios for increase in total mortality associated with this metric were even higher in the Northeast and Western United States (11% and 6.4% respectively) than the national average of 5.2%. Altogether, what our findings illustrate is that the spatiotemporal patterns of highs and lows for metrics related to heat in prison landscapes can greatly differ when examining raw air temperatures versus long‐term trends versus metrics of health risk.

Our analysis offers some similar and differing conclusions from the only prior national study of heat exposure and prisons (Tuholske et al., 2024). While there is some overlap between the states with the highest heat‐exposed carceral facilities found in both studies (California, Arizona, Texas, Oklahoma), the results from the prior study emphasize the Southeastern US more than our study does which is likely explained by the prior study's use of a metric that includes humidity which has a different spatial distribution of highs and lows than air temperature. While the inclusion of humidity can add value for characterizing heat exposure in certain conditions (Barnett et al., 2010), a national study on heat‐related mortality in prisons determined that neither heat index or wet bulb globe temperature better capture the relationship between heat and total mortality in the incarcerated population (Skarha et al., 2023). Tuholske et al. (2024) also report results as person‐days (exposure times population) which means that a facility could rank higher on their metric because of a high heat exposure or simply due to having a higher incarcerated population. Lastly, as their assessment does not disaggregate between types of carceral facilities, it is possible that their assessment of the most heat‐exposed states could be influenced by larger representations of non‐prison carceral facilities, whereas our study focuses on prisons. For the aforementioned reasons, our work is not directly comparable to Tuholske et al. (2024) and should be seen as complementary contributions to the nascent literature of the spatial patterns of heat exposure in carceral landscapes.

This study also offers a novel contribution on the population demographics of the most and least heat exposed prisons across the US. We found that the most heat‐exposed prisons have larger proportions of Hispanic and nonwhite populations than the least heat‐exposed prisons. The latter finding aligns with Glade et al. (2024) who found that carceral facilities in Colorado with higher rates of Black populations were more likely to be among facilities with the highest exposure to heat. While additional research would be needed to fully assess the relationship between heat exposure and prison demographics, and the drivers of those relationships, our results suggest that the patterns of inequitable heat exposure found in the general US by race/ethnicity (Benz & Burney, 2021; Harlan et al., 2006; Manware et al., 2022; Sayyed et al., 2024) may also be reflected in the US prison system. Addressing this issue will require addressing the criminalization of Black, Hispanic, and indigenous people and other marginalized identities that are incarcerated at higher rates (Mauer & King, 2007), which is likely contributing to this pattern of prisons with higher heat exposure having higher proportions of people of color.

Lastly, our paper makes two novel contributions regarding the study of heat as an environmental risk factor in prison settings: (a) we identified a set of measurable characteristics of a prison environment that we hypothesize could increase the incarcerated population's risk to the negative effects of heat and (b) we found evidence that prisons with any of nine facility‐level characteristics may experience increased risk to heat as a result of compounded vulnerability and exposure. For example, prisons with poor conditions related to staffing may not have the capacity to regularly implement heat‐intervention procedures such as distributing ice water or providing access to respite areas (Purdum et al., 2022). Or, in prisons that severely restrict movement, the incarcerated population may not be able to freely access cold showers to regulate body temperature. These vulnerabilities, coupled with the statistically significant higher heat exposure than their counterpart prisons without those conditions, may produce environments where the incarcerated population experience increased risk of adverse effects from heat in the absence of temperature regulation.

Taking all of the paper's contributions together, our study brings together distinct measures of exposure, vulnerability, and risk, which would each inform unique heat‐intervention strategies across the United States. An approach that considers risk in purely measures of high temperature exposure might prioritize the Southwestern US, whereas an approach that considers risk in relation to exposure *and* vulnerability might prioritize prisons in varied geographies with a particular set of facility‐level conditions (e.g., poor staffing). Environmental justice demands that all people have a right to live in environments free of hazards. Community leaders and decision makers should carefully consider which measures they want to apply as the choice of metric will have implications for who is included in fights for environmental justice.

### 4.1 Limitations

Our study has several limitations and uncertainties. While Daymet has strong agreement with weather station data, this modeled temperature data has uncertainties in the estimate of ambient air temperature that vary both in time and space, similar to other gridded climate data sets (P. E. Thornton et al., 2021). As our analysis largely utilizes time‐averaged temperatures, we hypothesize that the errors in Daymet would not significantly impact the relative rankings of the most heat‐exposed facilities or states. Despite this potential limitation, Daymet's high spatial resolution of 1 km provides insight into the spatiotemporal pattern of heat exposure in prison landscapes at a level of granularity not currently available in the literature. Another limitation of this study is that similar to prior literature (Tuholske et al., 2024), a full assessment of heat risk in prisons is also challenged due to incomplete availability of data on cooling mechanisms or indoor temperatures in prisons. While some of this information can be accessed through public records requests on a prison by prison or state by state basis, this research would greatly benefit from standardized laws requiring more accessible, transparent data about conditions of confinement. Further, in order to quantitatively assess whether facility‐level characteristics modifies heat risk, studies linking these characteristics to population‐level health outcomes would need to be performed, which is challenged by incomplete and inconsistent health outcome data across the prison system. As with most statistical analyses, the data used in the demographic and facility‐level characteristic analyses represent a sample of available records from the population of prisons. Thus, the relationships presented in the paper between these variables may not represent true population parameters. However, the missing data spanned 48 states and the District Columbia with no visible bias of missing data (Figure S2 in Supporting Information S1).

Future work should pursue diverse strategies for obtaining more data about heat, health, facility characteristics, and demographics in prisons to advance our understanding of the vulnerability of the incarcerated population to heat exposure. Future work should also extend these analyses to look at jails, immigrant detention facilities, and other carceral facilities in subset groups so as to understand similarities and differences in heat vulnerability faced across facility types subject to different regulatory contexts.

### 4.2 Broader Implications

When considering the multiple dimensions of heat exposure and risk in prisons presented in this paper, the natural conclusion might be that the government should mandate air conditioning in all prisons, which is an effective individual‐level intervention to mitigate heat exposure in the general population (Bouchama et al., 2007). However, this study does not advocate for unconditional implementation of air‐conditioning as the primary intervention to heat exposure and risk in prisons. The following quote from a man who was formerly incarcerated in prisons under the jurisdiction of the Texas Department of Criminal Justice well illustrates why an approach that centers air‐conditioning may be tenuous:

“I did like my last three years at Clemens [prison]. And Clemens has a nickname among the prison population that's been going around for probably like the last 50 or 60 years, and I call it ‘burn in hell, Clemens,’ because you are going to burn in there… They came up with this idea of ‘respite areas.’ If you felt too hot, you could go to respite and it was supposed to be like an air‐conditioned room. But I don't know what people's fallacies are about the [prison] guards and like, what they do and what they're capable of—But these people are like sadistic human beings… you could be on the verge of a heatstroke and [they're] not going to open your cell and escort you to respite. So they make them extremely inaccessible. It's really just the discretion of the guards and the discretion of the warden” (See Text S1 in Supporting Information S1 for source information).

A large body of literature details the way that many features of the contemporary prison system, including the exposure of the incarcerated population to environmental hazards, are not happenstance conditions but part of a larger function of punishment, neglect, and “death by design” (Alexander, 2012; Friedman, 2021; Pellow, 2021). There are numerous instances of carceral facilities purposefully being built on undesirable or “surplus” cheap land that are saturated with legacies of pollution and increasing climate risk (Bernd et al., 2017; Gilmore, 2007; Perdue, 2018; Ybarra, 2021). Attempts to remediate these hazards are hampered as formerly incarcerated people report witnessing frequent misuse of money for inadequate “repairs” to building conditions such as “putting tarps over holes in a roof” and facing retaliation from prison officials when trying to elevate concerns about prison conditions (Ovienmhada et al., 2023). Beyond physical environmental hazards, the COVID‐19 pandemic revealed inadequate capability of the prison system to mitigate public health hazards, resulting in dozens of prison systems with higher COVID‐19 infection and mortality rates than their respective states (Liu et al., 2022; Pyrooz et al., 2020; The Marshall Project, 2020). Some interventions that were implemented with the intent to curb the spread of COVID‐19 in prisons created heightened environments of isolation and poor mental health (Johnson et al., 2021). Collectively, these factors severely undermine the resilience of incarcerated individuals to protect their physical and mental health in the face of hazards (Friedman, 2021; Purdum et al., 2021).

As a consequence of these longstanding features of the prison system, many researchers and activists, including formerly incarcerated people who are at the forefront of advocacy against prison environmental injustice, support decarceration—the reduction of the number of incarcerated people and the discontinuation of carceral infrastructure—as an intervention to environmental‐related vulnerability in carceral settings (Ovienmhada et al., 2023; Purdum et al., 2021). A decarceration approach, which can be implemented through a variety of policy changes related to sentencing, pre‐trial detention, a moratorium on new prison infrastructure, and closure of prisons, would reduce the number of people in need of environmental protection, and enable the repurposing of the billions of dollars that would be needed to create truly humane conditions in the thousands of carceral facilities that exist in the US. This approach is already being championed by organizations such as Californians United for a Responsible Budget (CURB) and the Campaign to Fight Toxic Prisons (CFTP). Air‐conditioning, among other reforms, are interventions that could reduce short‐term heat‐related risk when implemented with additional policy guardrails. However, a decarceration approach is the most effective (physically and economically speaking) long‐term solution to reduce environmental‐related risk produced in carceral settings. In efforts to address the environmental injustices of the prison system, policy‐makers should consider these perspectives of people who have experiences living with environmental hazards in prisons or organizing against them, as their input can inform long‐term sustainable solutions that promote a healthier, more dignified populace.

## 5 Conclusion

This study examined spatiotemporal patterns of air temperature in US prison landscapes and facility or individual‐level characteristics of exposed prisons and incarcerated populations. Our study brings together distinct measures of exposure, vulnerability, and risk, which would each inform unique strategies for heat‐intervention in US prisons. Community leaders, policymakers, and decision makers should carefully consider which measures they want to apply, and include the voices of directly impacted people, as the differing metrics and perspectives will have implications for who is included in fights for environmental justice.

## Conflict of Interest

Ovienmhada and Wood report grants from NASA and grants from MIT's IDSS Initiative on Combating Systemic Racism during the conduct of the study.

## Supporting information

Supporting Information S1

## Data Availability

Data on the heat exposure and risk analysis are publicly available at Ovienmhada et al. (2024).

## References

[gh2569-bib-0001] Alexander, M. (2012). The New Jim Crow: Mass incarceration in the age of colorblindness. The New Press.

[gh2569-bib-0002] American Society of Heating, Refrigerating and Air‐Conditioning Engineers . (1997). 1997 ASHRAE Handbook: Fundamentals. ASHRAE. Retrieved from https://books.google.com/books?id=_zN_wgEACAAJ

[gh2569-bib-0003] Anno, B. , Graham, C. , Lawrence, J. , Shansky, R. , Bisbee, J. , & Blackmore, J. (2004). Correctional health care: Addressing the needs of elderly, chronically ill, and terminally ill inmates. Office of Justice Programs, Criminal Justice Institute. Retrieved from https://www.ojp.gov/ncjrs/virtual‐library/abstracts/correctional‐health‐care‐addressing‐needs‐elderly‐chronically‐ill

[gh2569-bib-0004] Barnett, A. G. , Tong, S. , & Clements, A. C. A. (2010). What measure of temperature is the best predictor of mortality? Environmental Research, 110(6), 604–611. 10.1016/j.envres.2010.05.006 20519131

[gh2569-bib-0005] Benmarhnia, T. , Deguen, S. , Kaufman, J. S. , & Smargiassi, A. (2015). Review article: Vulnerability to heat‐related mortality: A systematic review, meta‐analysis, and meta‐regression analysis. Epidemiology, 26(6), 781–793. 10.1097/EDE.0000000000000375 26332052

[gh2569-bib-0006] Benz, S. A. , & Burney, J. A. (2021). Widespread race and class disparities in surface urban heat extremes across the United States. Earth's Future, 9(7), e2021EF002016. 10.1029/2021EF002016

[gh2569-bib-0007] Bernd, C. , Loftus‐Farren, Z. , & Mitra, M. N. (2017). America’s toxic prisons: The environmental injustices of mass incarceration. Earth Island Journal, 32(2), 17–26.

[gh2569-bib-0008] Bouchama, A. , Dehbi, M. , Mohamed, G. , Matthies, F. , Shoukri, M. , & Menne, B. (2007). Prognostic factors in heat wave–related deaths: A meta‐analysis. Archives of Internal Medicine, 167(20), 2170–2176. 10.1001/archinte.167.20.ira70009 17698676

[gh2569-bib-0009] Bureau, U. C. (2024). 2019 National and State Population Estimates. Census.Gov. Retrieved from https://www.census.gov/newsroom/press‐kits/2019/national‐state‐estimates.html

[gh2569-bib-0010] Cardona, O. D. , Aalst, M. K. V. , Birkmann, J. , Fordham, M. , Gregor, G. M. , Rosa, P. , et al. (2012). Determinants of risk: Exposure and vulnerability. In Managing the Risks of Extreme Events and Disasters to Advance Climate Change Adaptation: Special Report of the Intergovernmental Panel on Climate Change (pp. 65–108). Cambridge University Press. 10.1017/CBO9781139177245.005

[gh2569-bib-0011] Carmichael, C. , Bickler, G. , Kovats, S. , Pencheon, D. , Murray, V. , West, C. , & Doyle, Y. (2012). Overheating and hospitals—What do we know? Journal of Hospital Administration, 2(1), 1. 10.5430/jha.v2n1p1

[gh2569-bib-0012] Cloud, D. H. , Williams, B. , Haardörfer, R. , Brinkley‐Rubinstein, L. , & Cooper, H. L. F. (2023). Extreme heat and suicide watch incidents among incarcerated men. JAMA Network Open, 6(8), e2328380. 10.1001/jamanetworkopen.2023.28380 37566416 PMC10422184

[gh2569-bib-0013] Colucci, A. R. , Vecellio, D. J. , & Allen, M. J. (2023). Thermal (In)equity and incarceration: A necessary nexus for geographers. Environment and Planning E: Nature and Space, 6(1), 638–657. 10.1177/25148486211063488

[gh2569-bib-0014] Enggist, S. , Møller, L. , & Galea, G. (2014). Prisons and health. Retrieved from https://www.who.int/europe/publications/i/item/9789289050593

[gh2569-bib-0015] Friedman, B. (2021). Toward a critical race theory of prison order in the wake of COVID‐19 and its afterlives: When disaster collides with institutional death by design. Sociological Perspectives, 64(5), 689–705. 10.1177/07311214211005485

[gh2569-bib-0016] Gaskin, C. J. , Taylor, D. , Kinnear, S. , Mann, J. , Hillman, W. , & Moran, M. (2017). Factors associated with the climate change vulnerability and the adaptive capacity of people with disability: A systematic review. Weather, Climate, and Society, 9(4), 801–814. 10.1175/WCAS-D-16-0126.1

[gh2569-bib-0017] Gilmore, R. W. (2007). Golden Gulag: Prisons, surplus, crisis, and opposition in globalizing California. University of California Press.

[gh2569-bib-0018] Glade, S. , Schmitz, C. , Barron, B. N. , Dashti, S. , Roudbari, S. , Liel, A. B. , et al. (2024). Hazards and incarceration facilities: Evaluating facility‐level exposure to floods, wildfires, extreme heat, and landslides in Colorado. Natural Hazards Review, 25(1), 04023047. 10.1061/NHREFO.NHENG-1556

[gh2569-bib-0019] Hajat, S. , & Kosatky, T. (2010). Heat‐related mortality: A review and exploration of heterogeneity. Journal of Epidemiology & Community Health, 64(9), 753–760. 10.1136/jech.2009.087999 19692725

[gh2569-bib-0020] Harlan, S. L. , Brazel, A. J. , Prashad, L. , Stefanov, W. L. , & Larsen, L. (2006). Neighborhood microclimates and vulnerability to heat stress. Social Science & Medicine, 63(11), 2847–2863. 10.1016/j.socscimed.2006.07.030 16996668

[gh2569-bib-0021] Holt, D. (2015). Heat in US prisons and jails: Corrections and the Challenge of Climate Change, Sabin Center for Climate Change Law, Columbia Law School. Retrieved from https://scholarship.law.columbia.edu/sabin_climate_change/124

[gh2569-bib-0022] Johnson, L. , Gutridge, K. , Parkes, J. , Roy, A. , & Plugge, E. (2021). Scoping review of mental health in prisons through the COVID‐19 pandemic. BMJ Open, 11(5), e046547. 10.1136/bmjopen-2020-046547 PMC8727680 33986064

[gh2569-bib-0023] Jones, T. S. , Liang, A. P. , Kilbourne, E. M. , Griffin, M. R. , Patriarca, P. A. , Wassilak, S. G. F. , et al. (1982). Morbidity and mortality associated with the July 1980 heat wave in St Louis and Kansas City, Mo. JAMA, 247(24), 3327–3331. 10.1001/jama.1982.03320490025030 7087075

[gh2569-bib-0024] Karmalkar, A. V. , & Bradley, R. S. (2017). Consequences of global warming of 1.5°C and 2°C for regional temperature and precipitation changes in the contiguous United States. PLoS One, 12(1), e0168697. 10.1371/journal.pone.0168697 28076360 PMC5226673

[gh2569-bib-0025] Kenny, G. P. , Flouris, A. D. , Yagouti, A. , & Notley, S. R. (2019). Towards establishing evidence‐based guidelines on maximum indoor temperatures during hot weather in temperate continental climates. Temperature, 6(1), 11–36. 10.1080/23328940.2018.1456257 PMC6422495 30906809

[gh2569-bib-0026] Kenny, G. P. , Yardley, J. , Brown, C. , Sigal, R. J. , & Jay, O. (2010). Heat stress in older individuals and patients with common chronic diseases. Canadian Medical Association Journal, 182(10), 1053–1060. 10.1503/cmaj.081050 19703915 PMC2900329

[gh2569-bib-0027] Li, M. , Gu, S. , Bi, P. , Yang, J. , & Liu, Q. (2015). Heat waves and morbidity: Current knowledge and further direction‐A comprehensive literature review. International Journal of Environmental Research and Public Health, 12(5), 5–5283. 10.3390/ijerph120505256 PMC4454966 25993103

[gh2569-bib-0028] Liu, Y. E. , LeBoa, C. , Rodriguez, M. , Sherif, B. , Trinidad, C. , del Rosario, M. , et al. (2022). COVID‐19 preventive measures in Northern California Jails: Perceived deficiencies, barriers, and unintended harms. Frontiers in Public Health, 10. 10.3389/fpubh.2022.854343 PMC9237366 35774562

[gh2569-bib-0029] Manware, M. , Dubrow, R. , Carrión, D. , Ma, Y. , & Chen, K. (2022). Residential and race/ethnicity disparities in heat vulnerability in the United States. GeoHealth, 6(12), e2022GH000695. 10.1029/2022GH000695 PMC9744626 36518814

[gh2569-bib-0030] Maresh, M. (2021). Prison vague on details of A/C outage. Imperial Valley Press Online. Retrieved from https://www.ivpressonline.com/news/local/prison‐vague‐on‐details‐of‐a‐c‐outage/article_7154a2be‐f807‐11eb‐9146‐fb354fff83ac.html

[gh2569-bib-0031] Maruschak, L. M. , & Buehler, E. D. (2021). Census of State and Federal Adult Correctional Facilities, 2019 – Statistical Tables. Bureau of Justice Statistics. Retrieved from https://bjs.ojp.gov/library/publications/census‐state‐and‐federal‐adult‐correctional‐facilities‐2019‐statistical‐tables

[gh2569-bib-0032] Mauer, M. , & King, R. S. (2007). Uneven justice: State rates of incarceration by race and ethnicity. The Sentencing Project. Retrieved from https://www.jstor.org/stable/resrep27349

[gh2569-bib-0033] Meadows, J. , Mansour, A. , Gatto, M. R. , Li, A. , Howard, A. , & Bentley, R. (2024). Mental illness and increased vulnerability to negative health effects from extreme heat events: A systematic review. Psychiatry Research, 332, 115678. 10.1016/j.psychres.2023.115678 38150812

[gh2569-bib-0034] Medina‐Ramón, M. , & Schwartz, J. (2007). Temperature, temperature extremes, and mortality: A study of acclimatisation and effect modification in 50 US cities. Occupational and Environmental Medicine, 64(12), 827–833. 10.1136/oem.2007.033175 17600037 PMC2095353

[gh2569-bib-0035] National Centers for Environmental Information . (2024). Climate at a Glance. National Time Series. National Centers for Environmental Information (NCEI). National Oceanic and Atmospheric Association. Retrieved from https://www.ncei.noaa.gov/access/monitoring/climate‐at‐a‐glance/national/time‐series/110/tavg/12/12/1895‐2023?base_prd=true&begbaseyear=1901&endbaseyear=2000

[gh2569-bib-0036] Nichols, A. W. (2014). Heat‐related illness in sports and exercise. Current Reviews in Musculoskeletal Medicine, 7(4), 355–365. 10.1007/s12178-014-9240-0 25240413 PMC4596225

[gh2569-bib-0037] Ovienmhada, U. , Diongue, A. , Pellow, D. N. , & Wood, D. (2023). Satellite remote sensing for environmental data justice: Perspectives from anti‐prison community organizers on the uses of geospatial data. Environmental Justice, 17(3), 181–192. 10.1089/env.2023.0019

[gh2569-bib-0038] Ovienmhada, U. , Krisch, M. , Trainor, C. , Diongue, A. , Hines, M. , West, A. , & Raychaudhuri, D. (2024). callmeufu/prison_environmental_justice: Prison environmental justice datasets (v2024‐2) [Dataset]. Zenodo. 10.5281/zenodo.13737458

[gh2569-bib-0039] Park, J. , Kim, A. , Kim, Y. , Choi, M. , Yoon, T. H. , Kang, C. , et al. (2024). Association between heat and hospital admissions in people with disabilities in South Korea: A nationwide, case‐crossover study. The Lancet Planetary Health, 8(4), e217–e224. 10.1016/S2542-5196(24)00027-5 38580423

[gh2569-bib-0040] Pellow, D. N. (2021). Struggles for environmental justice in US prisons and jails. Antipode, 53(1), 56–73. 10.1111/anti.12569

[gh2569-bib-0041] Perdue, R. T. (2018). Linking environmental and criminal injustice: The mining to prison pipeline in Central Appalachia. Environmental Justice, 11(5), 177–182. 10.1089/env.2017.0027

[gh2569-bib-0042] Prison Policy Initiative . (2024). 50 state incarceration profiles. Retrieved from https://www.prisonpolicy.org/profiles/

[gh2569-bib-0043] Purdum, C. , Dominick, A. , & Dixon, B. (2022). Extreme temperatures and Covid19 in Texas prisons. Texas A&M University, Hazard Reduction & Recovery Center. 10.13140/RG.2.2.25080.11522

[gh2569-bib-0044] Purdum, C. , Henry, F. , Rucker, S. , Williams, D. A. , Thomas, R. , Dixon, B. , & Jacobs, F. (2021). No justice, no resilience: Prison abolition as disaster mitigation in an era of climate change. Environmental Justice, 14(6), 418–425. 10.1089/env.2021.0020

[gh2569-bib-0045] Pyrooz, D. C. , Labrecque, R. M. , Tostlebe, J. J. , & Useem, B. (2020). Views on COVID‐19 from inside prison: Perspectives of high‐security prisoners. Justice Evaluation Journal, 3(2), 294–306. 10.1080/24751979.2020.1777578

[gh2569-bib-0046] Santucci, J. , Zarracina, J. , & Borresen, J. (2022). Map shows at least 44 states lack universal air conditioning in their prisons. USA Today. Retrieved from https://tangent.usatoday.com/in‐depth/graphics/2022/09/12/american‐prisons‐air‐conditioning‐heat‐climate/8017395001/

[gh2569-bib-0047] Sayyed, T. K. , Ovienmhada, U. , Kashani, M. , Vohra, K. , Kerr, G. H. , O’Donnell, C. , et al. (2024). Satellite data for environmental justice: A scoping review of the literature in the United States. Environmental Research Letters, 19(3), 033001. 10.1088/1748-9326/ad1fa4 PMC11457489 39377051

[gh2569-bib-0048] Skarha, J. , Dominick, A. , Spangler, K. , Dosa, D. , Rich, J. D. , Savitz, D. A. , & Zanobetti, A. (2022). Provision of air conditioning and heat‐related mortality in Texas prisons. JAMA Network Open, 5(11), e2239849. 10.1001/jamanetworkopen.2022.39849 36322085 PMC9631100

[gh2569-bib-0049] Skarha, J. , Spangler, K. , Dosa, D. , Rich, J. D. , Savitz, D. A. , & Zanobetti, A. (2023). Heat‐related mortality in U.S. state and private prisons: A case‐crossover analysis. PLoS One, 18(3), e0281389. 10.1371/journal.pone.0281389 36857338 PMC9976996

[gh2569-bib-0050] State of California . (2021). Capital Outlay Budget Change Proposal (COBCP)—Cover Sheet. Retrieved from https://esd.dof.ca.gov/Documents/bcp/2122/FY2122_ORG5225_BCP4500.pdf

[gh2569-bib-0051] Stephan, J. J. (2008). Census of state and federal correctional facilities, 2005. Bureau of Justice Statistics. Retrieved from https://bjs.ojp.gov/library/publications/census‐state‐and‐federal‐correctional‐facilities‐2005

[gh2569-bib-0052] Stivanello, E. , Chierzi, F. , Marzaroli, P. , Zanella, S. , Miglio, R. , Biavati, P. , et al. (2020). Mental health disorders and summer temperature‐related mortality: A case crossover study. International Journal of Environmental Research and Public Health, 17(23), 9122. 10.3390/ijerph17239122 33297344 PMC7731125

[gh2569-bib-0053] The Marshall Project . (2020). A state‐by‐state look at 15 months of coronavirus in prisons. The Marshall Project. Retrieved from https://www.themarshallproject.org/2020/05/01/a‐state‐by‐state‐look‐at‐coronavirus‐in‐prisons

[gh2569-bib-0054] Thornton, M. M. , Shrestha, R. , Wei, Y. , Thornton, P. E. , & Kao, S.‐C. (2022). Daymet: Annual climate summaries on a 1‐km grid for North America, version 4 R1. ORNL DAAC. 10.3334/ORNLDAAC/2130

[gh2569-bib-0055] Thornton, P. E. , Shrestha, R. , Thornton, M. , Kao, S.‐C. , Wei, Y. , & Wilson, B. E. (2021). Gridded daily weather data for North America with comprehensive uncertainty quantification. Scientific Data, 8(1), 190. 10.1038/s41597-021-00973-0 34301954 PMC8302764

[gh2569-bib-0056] Tuholske, C. , Lynch, V. D. , Spriggs, R. , Ahn, Y. , Raymond, C. , Nigra, A. E. , & Parks, R. M. (2024). Hazardous heat exposure among incarcerated people in the United States. Nature Sustainability, 7(4), 394–398. 10.1038/s41893-024-01293-y PMC12922630 41727418

[gh2569-bib-0057] Uejio, C. K. , Morano, L. H. , Jung, J. , Kintziger, K. , Jagger, M. , Chalmers, J. , & Holmes, T. (2018). Occupational heat exposure among municipal workers. International Archives of Occupational and Environmental Health, 91(6), 705–715. 10.1007/s00420-018-1318-3 29869703

[gh2569-bib-0058] US Department of Homeland Security . (2022). Prison boundaries. HIFLD Open Data. Retrieved from https://hifld‐geoplatform.hub.arcgis.com/

[gh2569-bib-0059] Venugopal, V. , Shanmugam, R. , & Kamalakkannan, L. P. (2021). Heat‐health vulnerabilities in the climate change context—Comparing risk profiles between indoor and outdoor workers in developing country settings. Environmental Research Letters, 16(8), 085008. 10.1088/1748-9326/ac1469

[gh2569-bib-0060] Vilher, L. (2017). Private prisons and the need for greater transparency: Private prison information act. Brooklyn Journal of Corporate, Financial & Commercial Law, 12(1), 213. Retrieved from https://brooklynworks.brooklaw.edu/bjcfcl/vol12/iss1/19

[gh2569-bib-0061] Vose, R. S. , Easterling, D. R. , Kunkel, K. E. , LeGrande, A. N. , & Wehner, M. F. (2017). Temperature changes in the United States. 10.7930/J0N29V45

[gh2569-bib-0062] Whitman, S. , Good, G. , Donoghue, E. R. , Benbow, N. , Shou, W. , & Mou, S. (1997). Mortality in Chicago attributed to the July 1995 heat wave. American Journal of Public Health, 87(9), 1515–1518. 10.2105/AJPH.87.9.1515 9314806 PMC1380980

[gh2569-bib-0063] World Population Review . (2024). Whitest States 2024. Retrieved from https://worldpopulationreview.com/state‐rankings/whitest‐states

[gh2569-bib-0064] Xu, Z. , FitzGerald, G. , Guo, Y. , Jalaludin, B. , & Tong, S. (2016). Impact of heatwave on mortality under different heatwave definitions: A systematic review and meta‐analysis. Environment International, 89–90, 193–203. 10.1016/j.envint.2016.02.007 26878285

[gh2569-bib-0065] Ybarra, M. (2021). Site fight! Toward the abolition of immigrant detention on Tacoma’s tar pits (and everywhere else). Antipode, 53(1), 36–55. 10.1111/anti.12610

