# Supplementary material for: Spatiotemporal Facility‐Level Patterns of Summer Heat Exposure, Vulnerability, and Risk in United States Prison Landscapes
Source: Geohealth. 2024 Sep 24;8(9):e2024GH001108. doi: 10.1029/2024GH001108 (PMC11421043; doi:10.1029/2024GH001108)
Supplement: Supplementary file 1 — Supporting Information S1 [file GH2-8-e2024GH001108-s001.docx]

*GeoHealth Journal*

Supporting Information for

**Spatiotemporal Facility-Level Patterns of Summer Heat Exposure, Vulnerability, and Risk in United States Prison landscapes**

Ufuoma Ovienmhada^1^, Mia Hines-Shanks^2^, Michael Krisch^3^, Ahmed T. Diongue^1^, Brent Minchew^4^, and Danielle R. Wood^5^

^1^Department of Aeronautics and Astronautics, Massachusetts Institute of Technology, Cambridge, Massachusetts, USA.

^2^Department of Computer Science, Grinnell College, Grinnell, Iowa, USA.

^3^Brown Institute for Media Innovation, Columbia University, New York, USA

^4^Department of Earth, Atmospheric, and Planetary Sciences, Massachusetts Institute of Technology, Cambridge, Massachusetts, USA.

^5^Space Enabled Research Group, Massachusetts Institute of Technology, Cambridge, MA, USA

Corresponding author: Ufuoma Ovienmhada (ufuoma@mit.edu )

**Contents of this file**

Text S1

Figures S1 to S2

Tables S1 to S6

**Introduction**

This Supporting Information provides the reader with additional figures and statistics tables which support the conclusions made in the Spatiotemporal, Demographic, and Facility-Level Patterns of Summer Heat Exposure in United States Prison landscapes manuscript. Figures S1 and S2 were created using QGIS. Tables S1 to S6 were created using statistics calculated in Python then formatted in Excel. The underlying data and the processing steps can be found at: 10.5281/zenodo.1234

**Text S1.** Narrative account obtained in interviews conducted by Author Ovienmhada under protocols approved by Massachusetts Institute of Technology's Institutional Review Board (IRB), the Committee on Use of Humans as Experimental Subjects (COUHES) pursuant to Federal regulations, 45 CFR Part 46.101(b)(2), protocol 2210000784


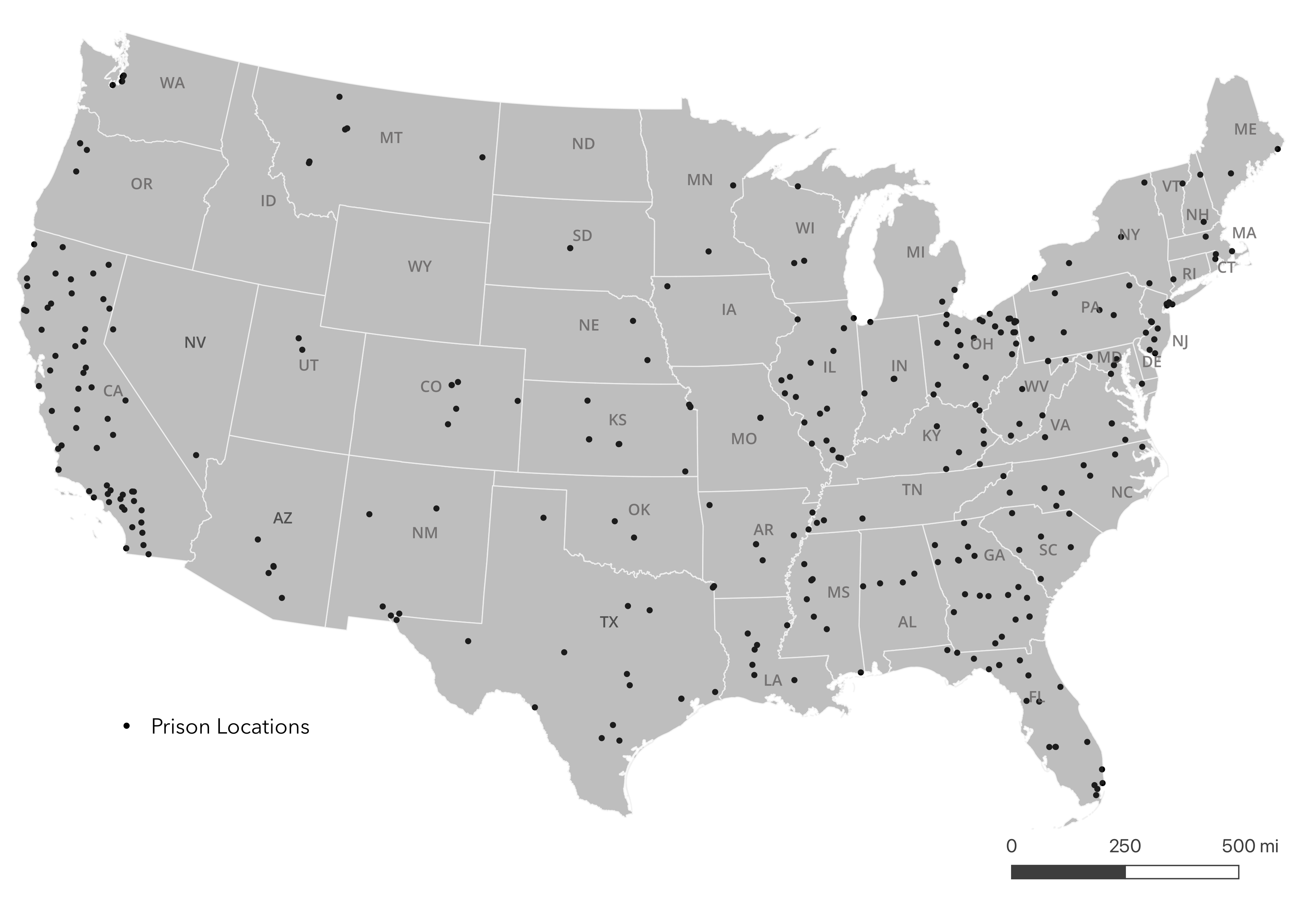
Figure S1. Point locations of prisons in HIFLD data that could not be matched to records in the BJSCensus2019 (n = 322). Missing data spanned 45 states and the District of Columbia. California had the highest share of missing records (n = 59, 17.4%)


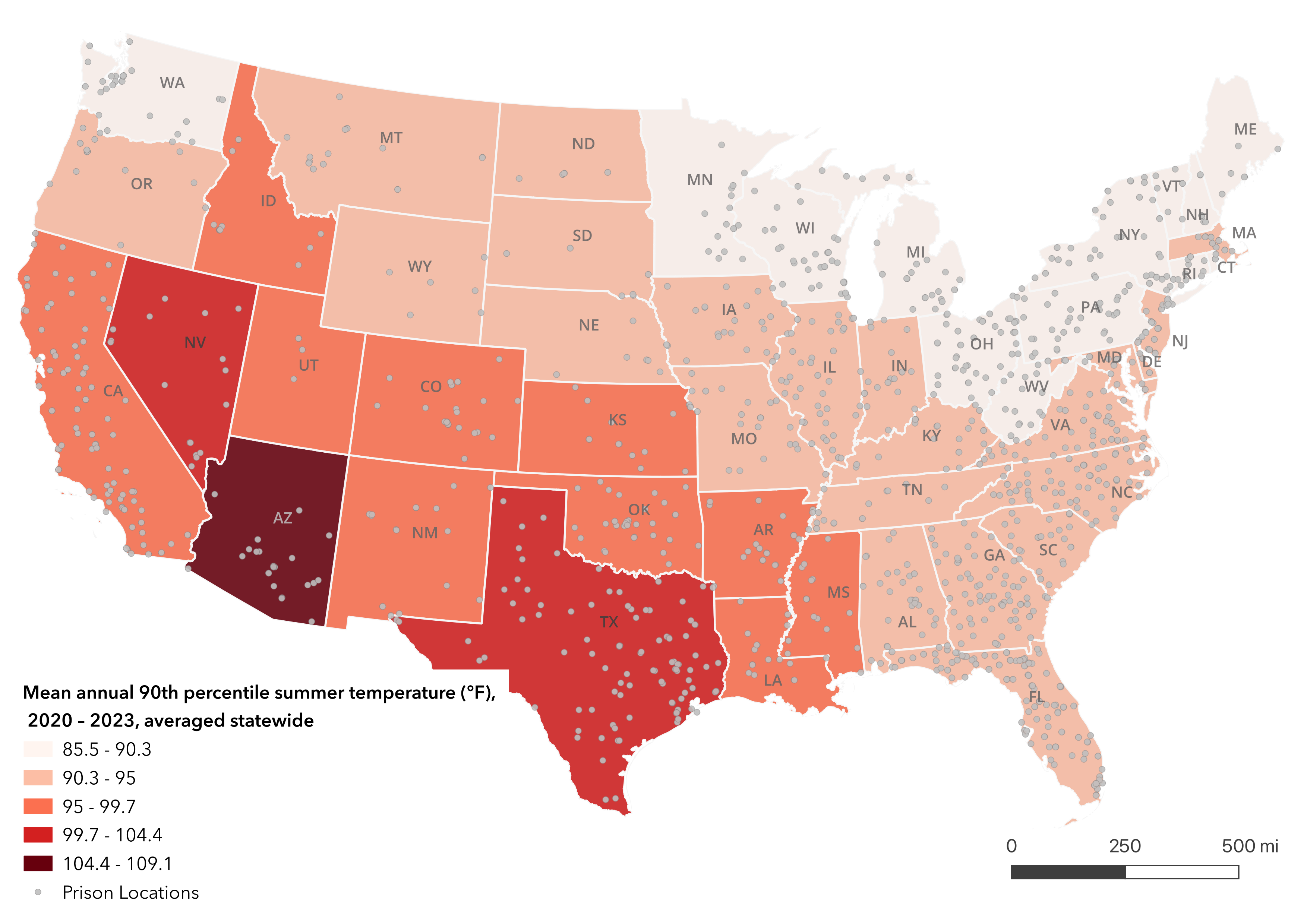
**Figure S2.** Mean annual 90^th^ percentile summer temperatures (°F) at prisons, 2020 – 2023, averaged by state. Circles show prison locations

Table S1. Summary of prison facility-level variables extracted from BJS Census 2019

| **Column Name** | **Response Type** |
| --- | --- |
| Security Level | Categorical |
| Major Facility Function: Geriatric Care | Yes/No |
| Major Facility Function: Medical Treatment | Yes/No |
| Major Facility Function: Mental Health Care | Yes/No |
| Major Facility Function: Alcohol/Drug Treatment | Yes/No |
| Geriatric Unit Present | Yes/No |
| Under Court Order for Conditions: Crowding | Yes/No |
| Under Court Order for Conditions: Mental Health Treatment | Yes/No |
| Under Court Order for Conditions: Medical Facilities | Yes/No |
| Under Court Order for Conditions: Accommodations of the Disabled | Yes/No |
| Under Court Order for Conditions: Staffing | Yes/No |
| Under Court Order for Conditions: Grievance Procedures | Yes/No |
| Under Court Order for Conditions: Totality of Conditions | Yes/No |
| Male Total | Count |
| Female Total | Count |
| Inmates Total | Count |
| White Inmates | Count |
| Black Inmates | Count |
| Hispanic Inmates | Count |
| American Indian Inmates | Count |
| Asian Inmates | Count |
| Native Hawaiian Inmates | Count |
| Total Staff | Count |
| Inmate Work Assignments in Farming/Agriculture | Yes/No |
| Inmates Housed in Restrictive Housing | Count |
| Inmates sentenced to more than one year | Count |
| Inmate Population Permitted to Leave | Categorical |

Table S2. Demographics for the prisons with the top and lowest 10 90^th^ percentile summer temperatures (°F), 2019

Table S3. Demographics for the prisons in the top and lowest decile (n = 126) of 90^th^ percentile summer temperature (°F), 2019

Table S4. 90^th^ percentile summer temperatures (°F), 2019, across all prisons population-weighted by race/ethnicity group

Table S5. Statistical test variables and results for facility-level heat risk analysis. Statistically significant results are in bold

Table S6. Statistical test variables and results for facility-level heat risk analysis continued. Statistically significant results are in bold

| **Variable** | **Factor Group Mean Temperatures (°F)** | | | **test** | **p-value** |
| --- | --- | --- | --- | --- | --- |
| Security Level | *Maximum/high* | *Medium* | *Minimum/low* | ANOVA | 0.171 |
|  | 93.3 | 92.7 | 93.5 |  |  |
| **Operator** | *Public* | *Private* |  | ANOVA | **0.025** |
|  | 92.8 | 94.9 |  |  |  |
| **Percentage of inmates permitted to leave facility** | *50% or more* | *Less than 50%* | *None* | Welch's ANOVA | **7.29E-08** |
|  | 91.6 | 91.8 | 93.3 |  |  |
